# Supplementary material for: VDR Regulates BNP Promoting Neurite Growth and Survival of Cochlear Spiral Ganglion Neurons through cGMP-PKG Signaling Pathway
Source: Cells. 2022 Nov 23;11(23):3746. doi: 10.3390/cells11233746 (PMC9739822; doi:10.3390/cells11233746)
Supplement: Supplementary file 1 [file cells-11-03746-s001.zip › cells-1988886-supplementary.pdf]

Table S1. The primers used for qRT-PCR experiments

Table 1.

Names	Sequences
BNP (rat)	Forward: 5′- GCTGCTGGAGCTGATAAGAGA-3′
	Reverse: 5′- CGCCGATCCGGTCTATCTTC-3′
NPRA (rat)	Forward: 5′- CCTTTCAGGCTGCCAAAAT -3′
	Reverse: 5′- ATCCTCCACGGTGAAGTTGA-3′
GAPDH (rat)	Forward: 5′-GAACGGGAAGCTCACTGG-3′
	Reverse: 5′-GCCTGCTTCACCACCTTCT-3′
VDR (human)	Forward: 5′- GTGGACATCGGCATGATGAAG-3′
	Reverse: 5′- GGTCGTAGGTCTTATGGTGGG-3′
Actin (human)	Forward: 5′- CTCCATCCTGGCCTCGCTGT -3′
	Reverse: 5′- GCTGTCACCTTCACCGTTCC-3′
BNP (human)	Forward: 5′- CAGCTTCTGCATCGTGGATT-3′
	Reverse: 5′-CATCTTCCTCCCAAAGCAGCC-3′

Table S2. The primers used for CHIP assays

Table 2.

	Sequences
H-NPPB-Site 1	Forward: 5′- ATCCTGTGTTGGCTTGGTGG-3′
	Reverse: 5′- TGAGAGATAGGGTCCAGTGATGA-3′
H-NPPB-Site 2	Forward: 5′- ATGGCGGATTGTGAGAGCAT-3′
	Reverse: 5′- CCGAGGGAACTTGGAGCAAA-3′

Luciferase assay promoter

> NPPB -Promoter-WT1

agatgaaggaggcactgggagaggggaaatccccatatctctggtatcccagcaaatagataaccatcattcc

agccatccttttgttttctttctttctttctttctttctttccttctttctttctttctttttcgctctctgtcaaccaggctgga

gtgcagtggcgtgatctcagctcactgcaacctccacctcctgggttcaagtgattctccttcctcagcctcccgag

tagctgggactacaggcgcctgccaccatgcccagctaatttttggtaattttagtagagacggggtttcaccgtg

gtctcgatctcctgacctcgtgatccgaccgcctcggcctctcaaagtgctgggattacaggcgtgaaccaccat

gcccagcctatccttttgttttccatcctgtgttggcttggtgggggagaggaggtgttgacacgtggaggacac

acatataaggcattcttgggtgacttcgtcatcactggaccctatctctcaaaattccagcgaaatctgc

> NPPB -Promoter-WT2

cccgttgctgtaaggactataagatggcggattgtgagagcatagggaaaggtctcggaggtctcttgtccttg

ctccacgcaggtctttctggcctgaaaatcccgttgaagagagcagctcttgagagtttgctccaagttccctcgg

ggtgatcagcaccacggacagcggccagggcgcccccgaggacccgcaggcaggcagggtgcacagcgg

cgagcaggtgctgcgctacgtgcgggccagggaactcgcgcggggaggggagaggcgccgcgggtggcg

gggtcttggccggggctgttttcgctgtgagtcaccccgtgctccccgcgctcacgtcggtcctcggaaagccgg

ggtcctccctgccttttccagcaacggtggggtggggaggcaggaagaaagcgccaacctaggacac

> NPPB -Promoter-MT1

Agatgaaggaggcactgggagaggggaaatccccatatctctggtatcccagcaaatagataaccatcattcc

agccatccttttgttttctttctttctttctttctttctttccttctttctttctttctttttcgctctctgtcaaccaggctgga

gtgcagtggcgtgatctcagctcactgcaacctccacctcccaggctgaagtgattctccttcctcagcctcccga

gtagctgggactacaggcgcctgccaccatgcccagctaatttttggtaattttagtagagacggggtttcaccgt

ggtctcgatctccctaccaggtgatccgaccgcctcggcctctcaaagtgctgggattacaggcgtgaaccacca

tgcccagcctatccttttgttttccatcctgtgttggcttggtgggggagaggaggtgttgacacgtggaggacac

acatataaggcattcttgggtgacttcgtcatcactggaccctatctctcaaaattccagcgaaatctgc

> NPPB -Promoter-MT2

Cccgttgctgtaaggactataagatggcggattgtgagagcatagggaaaggtctcggaggtctcttgtccttg

ctccacgcaggtctttctggcctgaaaatcccgttgaagagagcagctcttgagagtttgctccaagttccctcgg

ggtgatcagcaccacggacagcggccagggcgcccccgaggacccgcaggcaggcggagatcacagcggc

gagcaggtgctgcgctacgtgcgggccagggaactcgcgcggggaggggagaggcgccgcgggtggcgg

ggtcttggccggggctgttttcgctgtgagtcaccccgtgctccccgcgctcacgtcggtcctcggaaagccggg

gtcctccctgccttttccagcaacggtggggtggggaggcaggaagaaagcgccaacctaggacac

>R-Nppb-promoter

gctctaaagcacctcagtcaccttccccagcactggcggttatttttactgtgttagttgagatgagaagacccact

gtgagtgtccccactttatggatggggtcctggaccacataagtggagagagagagagaaagagagagaca

gacagacaggcagacagagacagagagagacagagagacagggacagagagacagacagagagagtc

agacagagacagagacagagacagagagacagagaggcagacagagagagacagagagagagagaga

gagagagagagagagagagagatgaattcactgctctctgcttcctgatggtggatgttacataaccaatggct

tcaagctcctgccacaggcaatgctgatagaagtgtagacagtgggaacctatctcagaggagagatgggatc

tatcaggaacttggctgggagccacttgtatgacatgttggccaagaatctgacttcattctgcctgtgtcctgag

aatttactgaaactgatgtggggatggtaggctaatttgttcctcagagggaaattgtgtgagaagggttaggg

gtttgagtgcttcgtactgggacttcaagtcttaaaaatgcaaattcacatacaagaaagctgcagctaagaatg

aagctgggggtggttctctgccccttgaaaacaatagcttaggagagagtgtgtctcccttgggtcagaacaca

gaacctcacataaacaggggtccaagaagccccgaggcttgcagaagaacctgacagctttctccaaatgtga

ctggtttggggctcatgaccttctaactatcccctttctctatctctaccttaacccactgccactcctggtcactgccc

ttgaccaacctgacccttgtgaacatagattctgcagaagggatagtgacattggccacacacctgggtgtatac

cccacagcctcatttcctcagagtctcatttctcacctgagtgggagactgggtgtgcgagggagaagtcactca

tggctgatcagctgagagctgggaagaggcccttcccacatgtctggtgtctaggcaccctttctctttcactcgt

ggtgtgggaggggacattggcacctggagatgccccagaaggcattgctgtgtgaccttgtctttgggccagg

atgaaaattctatcctaatcagctctcttttgttaaggggctggagttccagaagttccttgccatttaccagctttta

gtcatttgaatattggagagtgagcatctttgagctgaaatcttcacagggttttgaggggtggggagtactgga

aggtggggcagggtatcagagtggttgcgtgagccacctagctccttctggcctggagatcagtggagaaga

gaacttgaaagtttgtgtgaaggtcacgtgtggtgatcagaaccatggacagctaccagagtgcccggaagtg

gtgcagcccggccctacagctagatgcttggtagccgggcagggcctggattacaggttcgaggacactctcc

cagagaggctggtgatggtgggtgctaactggaagtgtttttgatgagtcaccccataagccccacacccgccc

caggctgggaaaactggggttctccctgtcatttccaatacgggtggggcacggtattaaaggccctcaggtttt

gcatgatagaagggagcaggaaacaaggacctgttagtgtcccccacacactcccaccctagcccagcccatg

cctatacaaggcctgcggtttatagcctccacccacaacgaacactccacagaggcacagaggggtgaattctc

aggtcctgagctcagcaggcaggaatgtgtctgataaatcagagataaccccacccctactccatgagaaggg

ctggccggacacccagccccaggataaaaggcagaggcaccgttgctgagaggctagtgcacgcgagctgct

tgcggag

(The highlighted part represents the mutation site)

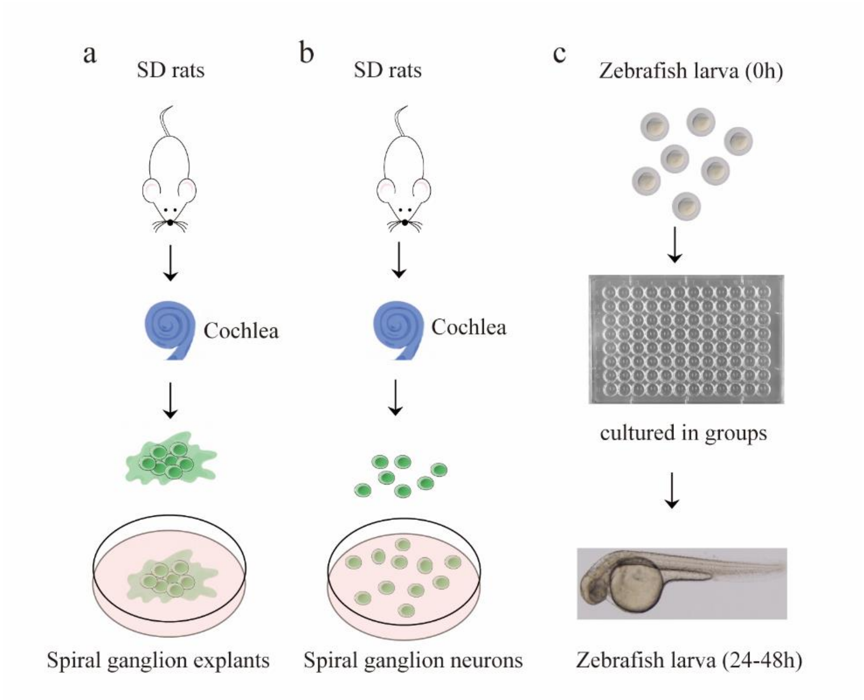

Figure S1. The diagram of the sequential experimentations. (a) The cochlea was removed from the P0 rat, and the middle turn of the spiral ganglions (SGs) was carefully separated from the spiral plate, cut into equal 300 to 500 μm sections, and transferred to 15 mm culture dishes. (b) The cochlea was removed from the P0 rat. Then, SGs were separated from the cochlea and enzymatically dissociated into single cells, culturing in 15 mm culture dishes**.** (c) Transparent larvae were isolated and randomly divided into different groups. The observation was made after incubation for 24-48 hours.

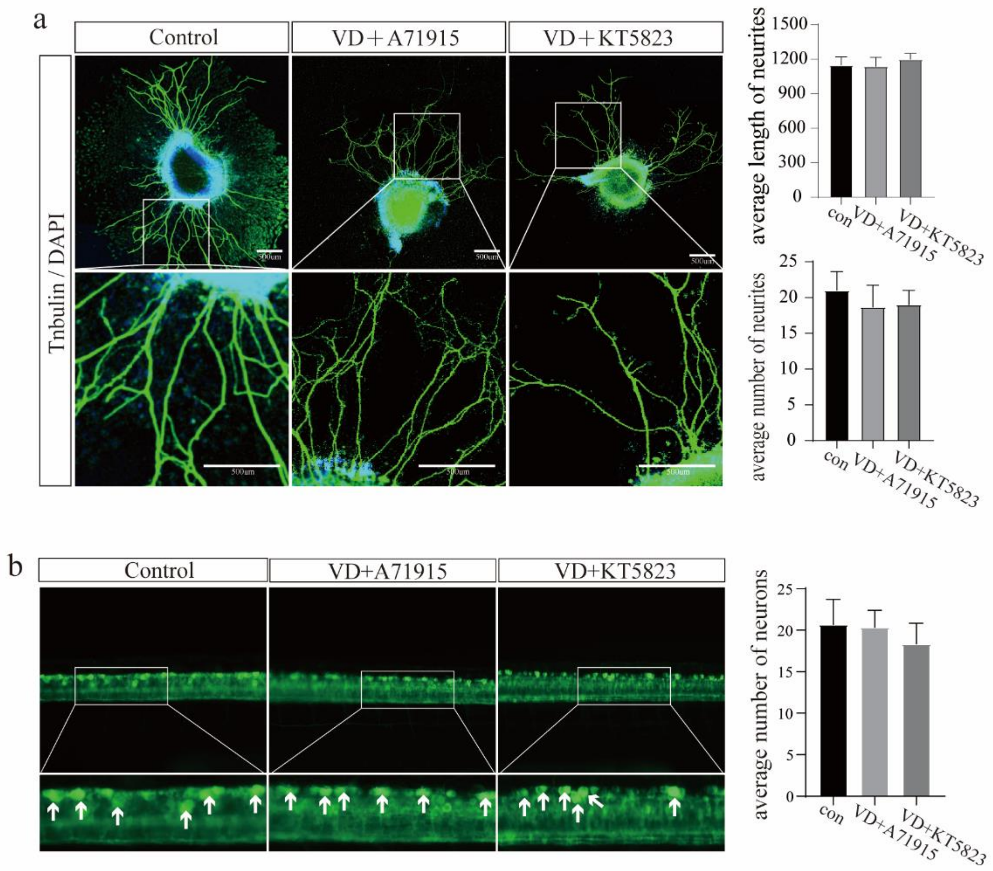

Figure S2. The inhibition of the GMP-PKG pathway stopped the pro-neurogenic effects of vitamin D. (a) SG explants were cultured in 10 nM vitamin D plus 1 μM NPR-A antagonist A71915, or 10 nM vitamin D plus 1 μM PKG inhibitor KT5823 followed by fluorescence immunostaining analysis. The results showed no significant difference in the number and length of neurites compared with the control group（b）Zebrafish larva was cultured in 10 nM vitamin D plus 1 μM A71915 and 10 nM vitamin D plus 1 μM KT5823. The results showed no significant difference in the number and length of neurites compared with the control group. Arrows indicate complete and countable neurons.
